# Effect of daily bathing with chlorhexidine on hospital-acquired bloodstream infection in critically ill patients: a meta-analysis of randomized controlled trials

**DOI:** 10.1186/cc14161

**Published:** 2015-03-16

**Authors:** E Choi, J Park

**Affiliations:** 1 Yeungnam University College of Medicine, Daegu, South Korea; 2 Uijeongbu St. Mary's Hospital, Uijeongbu, South Korea

## Introduction

Whole-body skin decolonization with chlorhexidine in critically ill patients reduces multidrug-resistant bacterial colonization, and catheter-related bloodstream infection (BSI). We performed a meta-analysis of randomized controlled trials to determine whether daily bathing with chlorhexidine decreased hospital-acquired BSIs in critically ill patients.

## Methods

We searched the MEDLINE, EMBASE, and Cochrane Central Register of Controlled Trials databases to identify randomized controlled trials that compared daily bathing with chlorhexidine and a control (daily bathing with soap and water or nonantimicrobial washcloths, or implementation of MRSA screening and isolation) in critically ill patients. The primary outcome was hospital-acquired BSIs. Secondary outcomes were adverse effects of chlorhexidine and the incidence of identified pathogens.

## Results

This meta-analysis included four studies. The overall incidence of hospital-acquired BSIs was significantly lower in the chlorhexidine group compared with the control 0.80 (95% CI, 0.71 to 0.90; *P *< 0.001; *I*^2 ^= 29.4%). Gram-positive (RR = 0.59, 95% CI, 0.44 to 0.79, *P *= 0.000; *I*^2 ^= 46.0%) and MRSA-induced (pooled RR = 0.64; 95% CI, 0.47 to 0.88, *P *= 0.006; *I*^2 ^= 0.0%) bacteremias were significantly less common in the chlorhexidine group. Chlorhexidine did not affect Gram-negative bacteremia or fungemia. The overall incidence of adverse events, such as skin rashes, was similar in both groups.

## Conclusion

Daily bathing with chlorhexidine was associated with a reduction in the rates of hospital-acquired BSI without significant complications in critically ill patients. It also decreased the incidence of Gram-positive hospital-acquired BSIs, especially MRSA.

